# Commentary: acupuncture-associated gastric mucosal repair and the EGF–EGFR-related signaling network: evidence, limitations, and prospects

**DOI:** 10.3389/fimmu.2026.2009774

**Published:** 2026-10-30

**Authors:** Wanting Liu, Weidong Shen

**Affiliations:** Department of Acupuncture, Shanghai Shuguang Hospital Affiliated to Shanghai University of Traditional Chinese Medicine, Shanghai, China

**Keywords:** acupuncture, EGF–EGFR signaling, electroacupuncture, epidermal growth factor receptor, gastric mucosal repair, moxibustion

## Introduction

We read with great interest the review by Jiang et al. (1), which summarizes the potential involvement of the epidermal growth factor (EGF)–epidermal growth factor receptor (EGFR) network in acupuncture-associated gastric mucosal repair and proposes a mechanistic evidence hierarchy spanning receptor activation, downstream signaling, and tissue repair. A major strength is the distinction between total EGFR expression, EGFR phosphorylation, and downstream signaling, together with the recognition that changes in extracellular signal-regulated kinase (ERK) or protein kinase B (Akt) alone do not establish an EGFR-dependent mechanism. The authors further note that integrated evidence linking phosphorylated EGFR (p-EGFR) activation and downstream signaling to tissue repair, with EGFR inhibition or blockade used to test causal involvement, remains lacking. We nevertheless believe that three issues merit further consideration: search coverage and currency of the evidence base, intervention-specific attribution of mechanistic evidence, and the distinction between EGFR dependence and EGF ligand specificity.

## Search scope and currency may affect the assessment of receptor-centered evidence

The review searched PubMed/MEDLINE, Web of Science, Embase, and the Cochrane Library through 15 December 2025, with additional reference-list screening (Section 2). The review did not report searches of Chinese bibliographic databases such as China National Knowledge Infrastructure (CNKI), Wanfang Data, the VIP Database for Chinese Technical Periodicals, and SinoMed. The review itself cites Chinese-language research, including a moxibustion study examining gastric EGFR and ERK1/2 (2) and a meta-analysis of acupuncture for peptic ulcer (3), illustrating the relevance of Chinese-language literature to this field. Although the inclusion of these studies does not establish that relevant evidence was missed, restricting the search to the reported databases may have reduced retrieval of older or non-indexed Chinese-language mechanistic studies.

For example, Li et al. (4) compared electroacupuncture at acupoints on the Foot-Yangming Stomach Meridian and Foot-Shaoyang Gallbladder Meridian in rats with stress-induced gastric mucosal injury. In the therapeutic electroacupuncture setting, gastric mucosal EGFR and EGFR messenger RNA (mRNA) expression were significantly higher in the Foot-Yangming Stomach Meridian group than in the model group (*p* < 0.01). Because p-EGFR was not assessed, this study does not establish receptor activation or EGFR-dependent causality, but it provides electroacupuncture-related evidence at the receptor-expression level. Additionally, two PubMed-indexed studies on electroacupuncture-related EGFR signaling reported that serum derived from electroacupuncture-treated rats enhanced EGFR gene expression in gastric mucosal cells (5), whereas the EGFR inhibitor PD153035 attenuated the associated downstream signaling changes (6). Although these studies did not establish a complete p-EGFR-centered causal chain, incorporating such receptor-expression and pharmacological-interference evidence could further enrich and refine the EGFR-related mechanistic evidence for electroacupuncture. Future updates could broaden the search to include Chinese bibliographic databases and provide more detailed reporting of database-specific search strategies, language restrictions, and study-selection procedures, thereby improving transparency and reproducibility.

Search currency also warrants clarification. The review was submitted in May 2026, revised in July 2026, and published in August 2026, with the literature search covering studies published through 15 December 2025. This Commentary was submitted on 25 September 2026. The published review does not state whether the search was updated during revision; therefore, such an update cannot be confirmed from the reported information. Given the limited number of receptor-centered studies, additional eligible evidence could affect the characterization of the available evidence across mechanistic levels. Explicitly reporting whether an updated search was conducted and, if so, its date and coverage would improve transparency and reproducibility, consistent with the literature-search domain of the Scale for the Assessment of Narrative Review Articles (SANRA) (7).

## Mechanistic evidence from different acupuncture-related interventions should be presented in a stratified manner

The original review grouped manual acupuncture, electroacupuncture, and moxibustion under the overarching framework of “acupuncture-associated interventions”, an approach that facilitates a holistic overview of gastric mucosal repair research related to acupuncture. However, the distribution of EGFR-related mechanistic evidence across these interventions is not uniform. The authors note that the direct receptor-related evidence identified in the review is primarily represented by a moxibustion study (2); meanwhile, additional studies (4–6) indicate that electroacupuncture-related evidence extends to gastric mucosal EGFR/EGFR mRNA expression and downstream signaling supported by evidence from pharmacological EGFR interference. Therefore, these interventions may occupy different positions across the mechanistic evidence hierarchy, including receptor expression, receptor activation, downstream signaling, and histological repair.

Although manual acupuncture, electroacupuncture, and moxibustion all fall under the umbrella of acupuncture-related interventions, their predominant modes of stimulation differ: mechanical stimulation for manual acupuncture, electrical stimulation superimposed on needling for electroacupuncture, and thermal stimulation for moxibustion. Future evidence frameworks could further annotate the intervention type and present relevant studies in a matrix of intervention type versus evidence level, thereby more accurately reflecting the current distribution of evidence for different acupuncture-related interventions within the proposed EGFR-related evidence hierarchy.

## EGFR dependence should be distinguished from EGF ligand specificity

The authors appropriately emphasize that total EGFR abundance is not equivalent to receptor activation and recommend assessing total EGFR, p-EGFR, the p-EGFR/EGFR ratio, downstream phosphorylation, repair outcomes, and, where feasible, EGFR inhibition or receptor blockade (Sections 4.2 and 7.1). We agree. However, a critical biological distinction remains: demonstrating an EGFR-dependent mechanism is not synonymous with proving an EGF-specific mechanism.

EGFR can be activated by several ligands besides EGF, including transforming growth factor-α (TGF-α), heparin-binding EGF-like growth factor (HB-EGF), amphiregulin, betacellulin, epiregulin, and epigen (8). Thus, even if future studies demonstrate increased EGFR phosphorylation after acupuncture and attenuation of mucosal repair after EGFR blockade, such findings would primarily support EGFR dependence; they would not, by themselves, establish EGF as the critical upstream ligand. To establish the ligand specificity of the EGF–EGFR axis, future studies could assess the temporal changes in EGF, TGF-α, HB-EGF, amphiregulin, and other EGFR ligands in the gastric mucosa. These assessments could be combined with ligand-neutralizing antibodies, ligand knockout, or conditional interventions to evaluate the effects of ligand perturbation on EGFR phosphorylation, downstream signaling, and mucosal repair under appropriately controlled conditions, rather than relying solely on EGFR inhibition.

## Discussion

In summary, Jiang et al. (1) provide a useful framework for studying acupuncture-associated gastric mucosal repair. We suggest that the evidence base be reassessed through a broader and updated search, that mechanistic findings be classified according to intervention modality and level of evidence, and that EGFR dependence be distinguished from EGF ligand specificity. These refinements may provide a more accurate evidence map and more precise priorities for future research.
